# Molecular mechanism of Tembusu virus nonstructural protein 5 antagonising RNA interference

**DOI:** 10.1186/s13567-026-01731-z

**Published:** 2026-04-09

**Authors:** Meijuan Zhang, Siming Zhu, Chengguang Lu, Yafei Qin, Saisai Zhao, Xinhui Wei, Mingtian Mao, Bing Li, Xinyuan Xu, Mian Wu, Huihui Li, Zhuo Zhang, Youxiang Diao, Dalin He, Tang Yi

**Affiliations:** 1 Institute of Animal Science, Chinese Academy of Agricultural Sciences, No. 2, Yuanmingyuan West Road, Beijing, 100091 China; 2 College of Veterinary Medicine, Shandong Agricultural University, No. 7, Panhe Street, Tai’an, 271001 Shandong China

**Keywords:** Tembusu virus, nonstructural Protein 5, innate immunity, RNA interference, viral suppressor of RNAi

## Abstract

Tembusu virus (TMUV), a member of the genus *Orthoflavivirus* within the family *Flaviviridae*, is an emerging pathogen that severely impacts waterfowl, causing decreased egg production in laying ducks and neurological symptoms in ducklings and growing birds. RNA interference (RNAi) is a conserved post-transcriptional regulatory mechanism mediated by small non-coding RNAs in eukaryotic cells, while many viruses can antagonise host RNAi-mediated antiviral immunity by encoding viral suppressors of RNAi (VSRs). In this study, we demonstrated that TMUV antagonises the RNAi pathway. Using enhanced green fluorescent protein (EGFP)-based RNAi reporter systems in both HEK293T and *Drosophila* S2 cells, we identified the TMUV nonstructural protein 5 (NS5) as a potent VSR. Furthermore, we found that NS5 not only interacts with DEAD-box helicase 3 X-linked (DDX3X), a key protein in the RNAi pathway, but also binds directly to double-stranded RNA (dsRNA). In summary, our findings indicated that TMUV NS5 can act as a VSR in vitro, thereby providing a theoretical foundation for the development of antiviral therapeutics.

## Introduction

Tembusu virus (TMUV) is a member of the *Orthoflavivirus* genus in the *Flaviviridae* family [1]. Viruses within the same genus include Japanese encephalitis virus (JEV), West Nile virus (WNV), yellow fever virus (YFV), and Zika virus (ZIKV), etc. [1]. The host range of TMUV is vast, and it can infect multiple varieties of ducks, including Peking duck, Shan Partridge duck, Jinding duck, Shaoxing duck, Jinyun Partridge duck and wild duck. In addition, it demonstrates cross-species infectivity in chickens, geese, pigeons, sparrows, mosquitoes and murine models [2]. The clinical manifestations after viral infection include systemic pathology targeting the spleen, bursa of Fabricius, thymus, ovaries and brain [3]. TMUV infection causes can lead to a decrease in egg production of laying ducks, whereas ducklings and growing ducks may exhibit neurological symptoms. The incidence rate can reach 100%, with mortality rates ranging from 5% to 30%.

TMUV is a single-stranded positive-sense RNA virus, with a genome length of 10 990 nt, encoding a polyprotein precursor. This polyprotein is cleaved and processed by host proteases into ten mature viral proteins, including three structural proteins: the capsid protein (C), the pre-membrane protein (prM), and the envelope glycoprotein (E), as well as five nonstructural proteins: NS1, NS2A, NS2B, NS3, NS4A, NS4B and NS5. Among these, the NS5 protein represents the largest nonstructural protein implicated in numerous processes that are pivotal to the virus’s life cycle. These processes include the formation of the 5′ cap, the initiation of viral RNA synthesis and the regulation of viral replication and translation [4–6].

RNA interference (RNAi) is a highly conserved gene silencing mechanism mediated by small non-coding RNAs, which is widely present in various eukaryotic organisms in plants, fungi, invertebrates and mammals. It is an important antiviral immune defence mechanism [7, 8]. When a virus, especially a positive-stranded RNA virus, invades a host cell, the double-stranded RNA (dsRNA) produced during its replication serves as a pathogen-associated molecular pattern (PAMP) and can be recognised and processed by the Dicer protein into a large amount of viral small interfering RNA (vsiRNA) [9]. These vsiRNAs are structurally similar to endogenous small RNAs and bind to host Argonaute (AGO) proteins to form RNA-induced silencing complexes (RISCs), thereby specifically recognising and binding to the target mRNA of the virus, mediating its silencing or degradation, and effectively inhibiting virus replication [10, 11].

However, numerous viruses can counteract the host’s natural RNAi immunity by encoding viral suppressors of RNAi (VSR). For instance, the serine protease and accessory proteinase of Potato virus Y (PVY) [12], the Tat protein of human immunodeficiency virus (HIV) [13], the N protein of severe acute respiratory syndrome coronavirus 2 (SARS-CoV-2) [14], the capsid protein of Semlik Forest virus (SFV) [15] and the 3A protein of Coxsackievirus B3 (CVB3) [16] are all VSR proteins, which can effectively counteract the host’s RNAi effect. Furthermore, many viruses of the genus *Flavivirus* can also antagonise RNAi activity by encoding VSR to ensure their survival and proliferation. Such as the NS2A protein of Japanese encephalitis virus (JEV), the sfRNA of West Nile virus (WNV) [17], the NS2A and NS4B protein of the dengue virus (DENV) [18, 19] and the envelope protein 2 and core protein of the hepatitis C virus (HCV) [20]. TMUV is also a member of the *Flavivirus* genus, but whether it encodes a functional VSR remains elusive.

In this study, we demonstrated that TMUV infection of mammalian cells induced robust production of vsiRNAs, thereby activating the RNAi antiviral immune pathway. Furthermore, as the infection progressed, TMUV antagonised RNAi. Subsequently, we identified that the NS5 protein of TMUV is a potent VSR. The NS5 protein suppressed RNAi through dual mechanisms: direct binding to dsRNA and interaction with DEAD-box helicase 3 X-linked (DDX3X), a core component of the RNAi pathway. Overall, our findings demonstrated that TMUV NS5 possesses VSR activity, thereby providing potential targets for the development of novel antiviral preparations based on RNAi.

## Materials and methods

### Plasmids and RNAs

For the reversal-of-silencing assay in mammals, the plasmid pEGFP-C1 was used to express the enhanced GFP (EGFP) protein. For the expression of DDX3X, FB2, TMUV NS5 and its other proteins in mammalian cells, their open reading frame (ORFs) were cloned into the vector pCAGGS-HA. To express proteins in *Drosophila* S2 cells, the ORFs were constructed into the insect expression vector pLB-3×HA, respectively. For the purification of the GST fusion NS5 protein, its ORF was inserted into the pGEX-6p-1 vector. The anti-EGFP siRNA and the plasmid pGPU6-Hygro-GFP-274 short hairpin RNA (shRNA) were chemically synthesised by Ji Ma, Shanghai, China.

### Cell culture and transfection

HEK293T cells were maintained in Dulbecco’s modified Eagle medium (DMEM) supplemented with 10% fetal bovine serum (FBS; HyClone, Logan, UT) and 1% penicillin–streptomycin at 37 ℃ in an incubator with 5% CO_2_. The *Drosophila* S2 cell line was kindly provided by Zizhang Zhou (Taian, Shandong, China). *Drosophila* S2 cells were cultured in SFX-Insect medium (HyClone, Logan, UT) with 10% FBS at 27 ℃. Before transfection with Lipofectamine 2000 Reagent (Thermo Fisher Scientific, Waltham, USA), the medium was changed to DMEM or SFX-Insect medium containing 2% FBS without any antibiotics.

HEK293T or S2 cells were seeded at 1 × 10^6^ cells per well (6-well plates) or 5 × 10^5^ cells per well (12-well plates). Then, transfection was carried out with Lipofectamine 2000 Reagent (Thermo Fisher Scientific, Waltham, USA) according to the manufacturer’s instructions, using a reagent-to-plasmid DNA ratio of 3:1 (μL:μg).

### Virus infection

TMUV (strain TC2B) was from our laboratory and amplified in C6/36 cells. On the day of infection, the medium was changed to 2% FBS–DMEM, and then the viruses were added to HEK293T cells at a multiplicity of infection (MOI) of 5. Total RNAs were extracted at 12 and 24 h post-infection (hpi) and subjected to deep sequencing analysis of small RNAs.

### Quantitative real-time PCR

Quantitative real-time PCR (qRT-PCR) using MagicSYBR Mixture (Cwbio, Beijing, China) was carried out to detect the expression of *EGFP* mRNA and *GAPDH* mRNA. Data represent means and standard deviations (SD) from three repeated experiments.

### Deep sequencing and data analysis

Briefly, the library sequencing was performed on the Illumina HiSeq 2000 platform at Majorbio (Shanghai, China). Raw reads were processed using Majorbio in-house software and Fastx-Toolkit to obtain high-quality clean reads, following these steps: (i) trimming bases with a quality value < 20 at the 3′ end, (ii) removing adapter sequences and reads without insert fragments (resulting from adapter self-ligation), (iii) excluding reads with an N content exceeding 10%, (iv) discarding reads shorter than 18 nt and (v) retaining sRNA sequences with lengths ranging from 18 to 32 nt for subsequent analysis.

To identify viral small interfering RNAs (vsiRNAs), the clean reads were mapped to the TMUV genome (GenBank: MH764605.1) using Blastn software with up to two nucleotide mismatches allowed. The vsiRNA reads were then counted and normalised to the total number of vsiRNA reads to generate counts per million (CPM) values, which were used to analyse the size distribution and abundance of vsiRNAs of different lengths. Specifically, the distribution of 21–24 nt vsiRNAs across the viral genome was visualised on the basis of their mapped read counts. All small RNA sequencing experiments were performed in three independent replicates.

### Co-immunoprecipitation

For immunoprecipitation (IP), transfected HEK293T cells were harvested in IP lysis buffer (Beyotime Biotechnology, Shanghai, China), and protease inhibitor cocktail (Solarbio, Beijing, China), followed by IP using an HA-Tag antibody or rabbit IgG (Proteintech, Rosemont, IL, USA) and Protein A + G agarose beads (Beyotime Biotechnology, Shanghai, China) according to the manufacturer’s instructions. Briefly, cells were lysed at 4 °C for 25 min. Lysates were clarified at 12 000 rpm/min for 10 min at 4 °C, and then the lysates were pre-cleared by incubation with protein A + G agarose beads at 4 °C for 2 h. Then, the pre-cleared lysates were incubated with antibodies (anti-HA or anti-IgG) and protein A + G agarose beads at 4 ℃ for 16 h. The antibody-bound complexes were washed five times with the same lysis buffer. Finally, proteins were extracted from the complexes and analysed by western blotting.

### Western blotting

Cells were harvested in western blotting lysis buffer (Beyotime Biotechnology, Shanghai, China) and a protease inhibitor cocktail (Solarbio, Beijing, China). The lysates were then subjected to 10% SDS–PAGE and western blotting. The antibodies used in this study included anti-tubulin (1:100 000), anti-HA (1:5000), anti-GFP (1:3000) and anti-GST (1:10 000), which were purchased from Proteintech, Rosemont, IL, USA. In addition, the TMUV NS5 protein monoclonal antibody was prepared and stored in our laboratory.

### Silver staining and mass spectrometry identification

Following SDS–PAGE, the gels were stained using a rapid silver staining kit (Beyotime Biotechnology, Shanghai, China) according to the manufacturer’s instructions. Then, protein bands exhibiting differential expression between the control and experimental group were excised from the gels. The excised protein bands were subsequently analysed by liquid chromatography–tandem mass spectrometry (LC–MS) for protein identification by Shanghai Luming Company.

### GST pull-down

The pGEX-6p-1-NS5 and pGEX-6p-1 (empty vector control) plasmids were transformed into *Escherichia coli* BL21 (DE3). Transformed cells were grown to mid-log phase (OD_600_ ≈ 0.6) at 37 ℃ in LB medium containing ampicillin (100 µg/mL). Protein expression was then induced by the addition of IPTG to a final concentration of 0.8 mM, followed by incubation at 16 °C for 18 h with shaking. The bacterial solution was centrifuged at 4000 rpm/min for 10 min and resuspended in phosphate-buffered saline (PBS). Then, the bacterial suspension was sonicated on ice. The expression levels of recombinant GST-NS5 fusion protein (pGEX-6p-1-NS5) and GST control protein (pGEX-6p-1) in whole cells, supernatants and precipitates were determined by western blotting.

The GST-NS5 and GST proteins were purified using the GST Protein Purification Kit (Mdbio, Qingdao, China) according to the manufacturer’s instructions. The GST pull-down assay was performed using a GST pull-down kit (Fitgene, Guangzhou, China) to detect whether there is an interaction between the GST-NS5 or GST proteins and the HA-DDX3X proteins. Briefly, the purified supernatants of the GST-NS5 or GST protein were incubated with resin at 4 ℃ for 2 h. The complex was washed three times with wash buffer. The HA-DDX3X protein was incubated with the complex at 4 ℃ for 16 h. Then, the new complex was washed with the same wash buffer three times and detected by western blot analysis.

### Preparation of EGFP-dsRNA

The DNA template was generated by PCR amplification using the pEGFP-C1 plasmid as template with primers T7-EGFP forward (5′-TAATACGACTCACTATAGGGATGGTGAGCAAGGGCGAGGA-3′) and T7-EGFP reverse (5′-TAATACGACTCACTATAGGGTAGGTCAGGGTGGTCAC-GAG-3′). The PCR product was electrophoresed on a 1.0% agarose gel and purified using a gel extraction kit (Omega Bio-tek, Norcross, GA) according to the manufacturer’s instructions. The purified product served as the template for subsequent in vitro transcription. Then, in vitro transcription of EGFP-dsRNA was performed using the T7 RNAi Transcription Kit (Vazyme, Nanjing, China) according to the manufacturer’s instructions.

### RNA- immunoprecipitation

The RNA-IP assay was performed using the RNA IP Kit (Geneseed, Guangzhou, China) according to the manufacturer’s instructions. Briefly, after transfection for 48 h, HEK293T cells were lysed in 1× Buffer A containing 1% RNase inhibitor and a protease inhibitor cocktail at 4 ℃ for 10 min. After centrifugation for 10 min at 12 000 rpm/min, the supernatant was pre-cleared by incubation with protein A + G beads at 4 °C for 10 min. Then the beads were pre-cleared with 1× Buffer A and Buffer D. The pre-cleared beads were incubated with antibodies (anti-HA or anti-IgG as a negative control) at 4 °C for 2 h. The complex was used to capture the antigen by incubation with the pre-cleared supernatant. The antibody-bound complexes were washed eight times with 1× Buffer B. Finally, RNAs were extracted using Trizol reagent (TransGen Biotech, Beijing, China).

## Results

### TMUV antagonises the RNAi antiviral immune pathway in HEK293T cells

**Figure 1:**
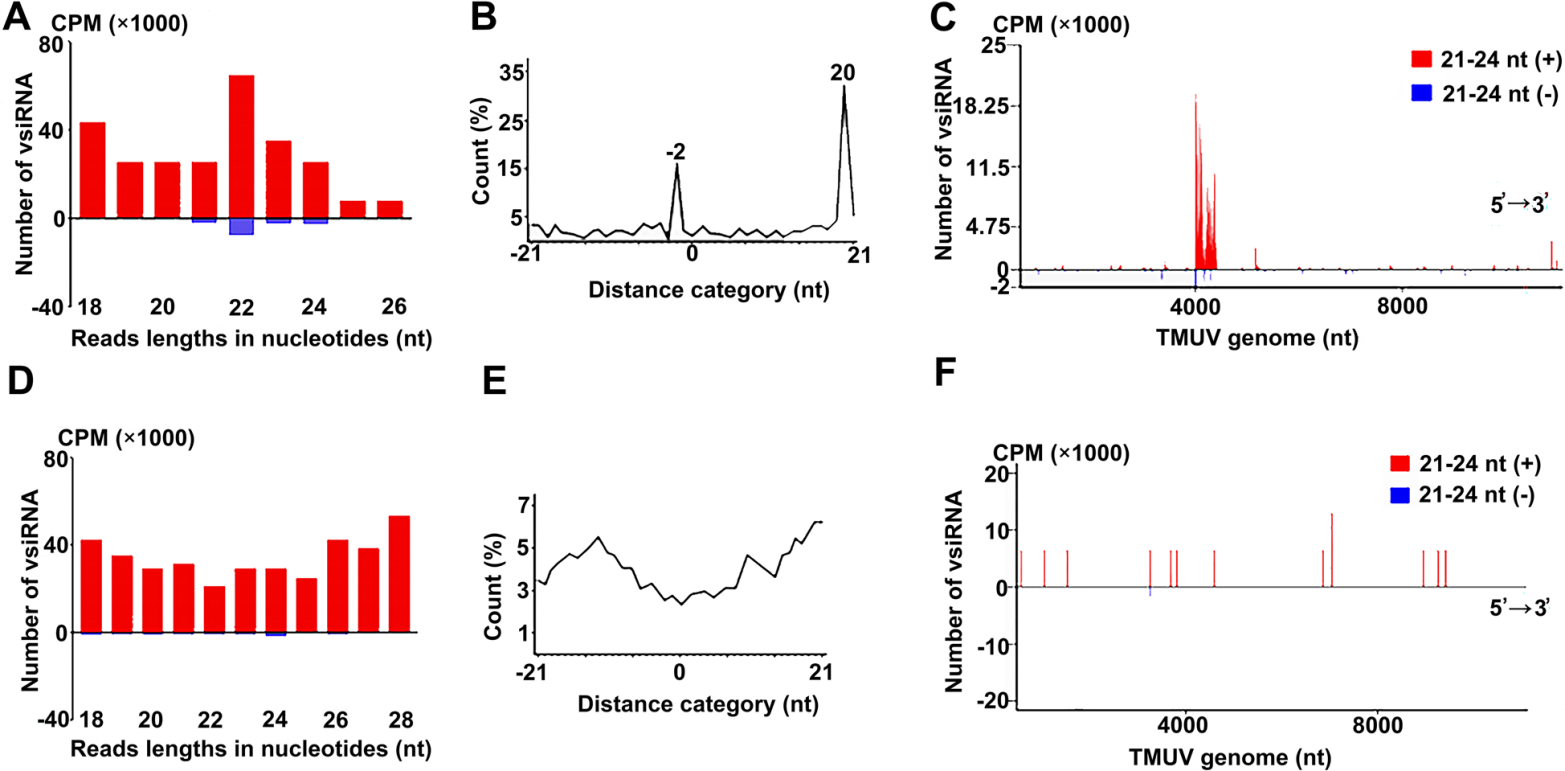
**Charactersation of vsiRNA induced by TMUV infection in HEK293T cells.** **A**–**C** and **D**–**F** depict the characteristic features of vsiRNA induced at 12 hpi and 24 hpi, respectively. **A** and **D** The size distribution and abundance (counts per million of total viral reads [CPMs]) of total vsiRNAs of indicated samples. Red represents positive-strand vsiRNA fragments; blue represents negative-strand vsiRNA fragments. **B** and **E** The proportion of each complementary paired vsiRNA pair among the 22 nt sized vsiRNAs. The axis label “Distance category (nt)” refers to the complementary pairing distance of vsiRNAs. The ‘−2’ peak represents the proportion of vsiRNA with complete 20 nt base pairing and a 2 nt overhang at the 3′ end. **C** and **F** The positional distribution and quantity of 21–24 nt sized vsiRNA on the TMUV genome (the *x*-axis TMUV genome ‘nt’ indicates the positive/negative strand direction of the TMUV genome, with 5′ → 3′ corresponding to the [+] strand). Red represents positive-strand vsiRNA fragments; blue represents negative-strand vsiRNA fragments.

TMUV not only replicates in mosquito cells C6/36 but also replicates in mammalian cells, such as HEK293 and HEK293T, and causes cytopathic effects. Therefore, this study established a TMUV (MOI = 5) infection system using HEK293T cells as a model. At 12 and 24 hpi, total cellular RNA was extracted and subjected to small-RNA deep sequencing to assess whether viral infection elicits vsiRNAs production and activates the RNAi antiviral pathway. The results showed that distinct vsiRNAs were observed in HEK293T cells at 12 hpi (Figures 1A–C). Although the abundance of antigenomic (−)-vsiRNA reads was relatively lower compared with that of genomic (+)-vsiRNA reads, vsiRNAs of 22 ± 1 nt are clearly accumulated in both polarities, with a peak at 22 nt (Figure 1A). These vsiRNAs were enriched for a population at 22 nt for 20 nt perfectly base-paired duplexes with 2 nt 3′-overhangs, termed peak ‘2’, demonstrating that a significant proportion of these TMUV-derived vsiRNAs display canonical siRNA properties (Figure 1B). Moreover, the genomic origin analysis of the 22 ± 1 nt vsiRNA reads showed that these TMUV-derived vsiRNAs were highly concentrated around the 4000-nt position of the TMUV genome (Figure 1C). In contrast, vsiRNAs produced by cells 24 h post-TMUV infection did not exhibit the characteristic features of vsiRNAs in terms of size, distribution and genomic position (Figures 1D–F). These results indicate that TMUV infection activates the host RNAi-mediated antiviral immunity; however, as the infection time continues, TMUV antagonises the host RNAi antiviral immune pathway.

### Establishment of the siRNA-mediated EGFP-RNAi system in HEK293T cells

**Figure 2:**
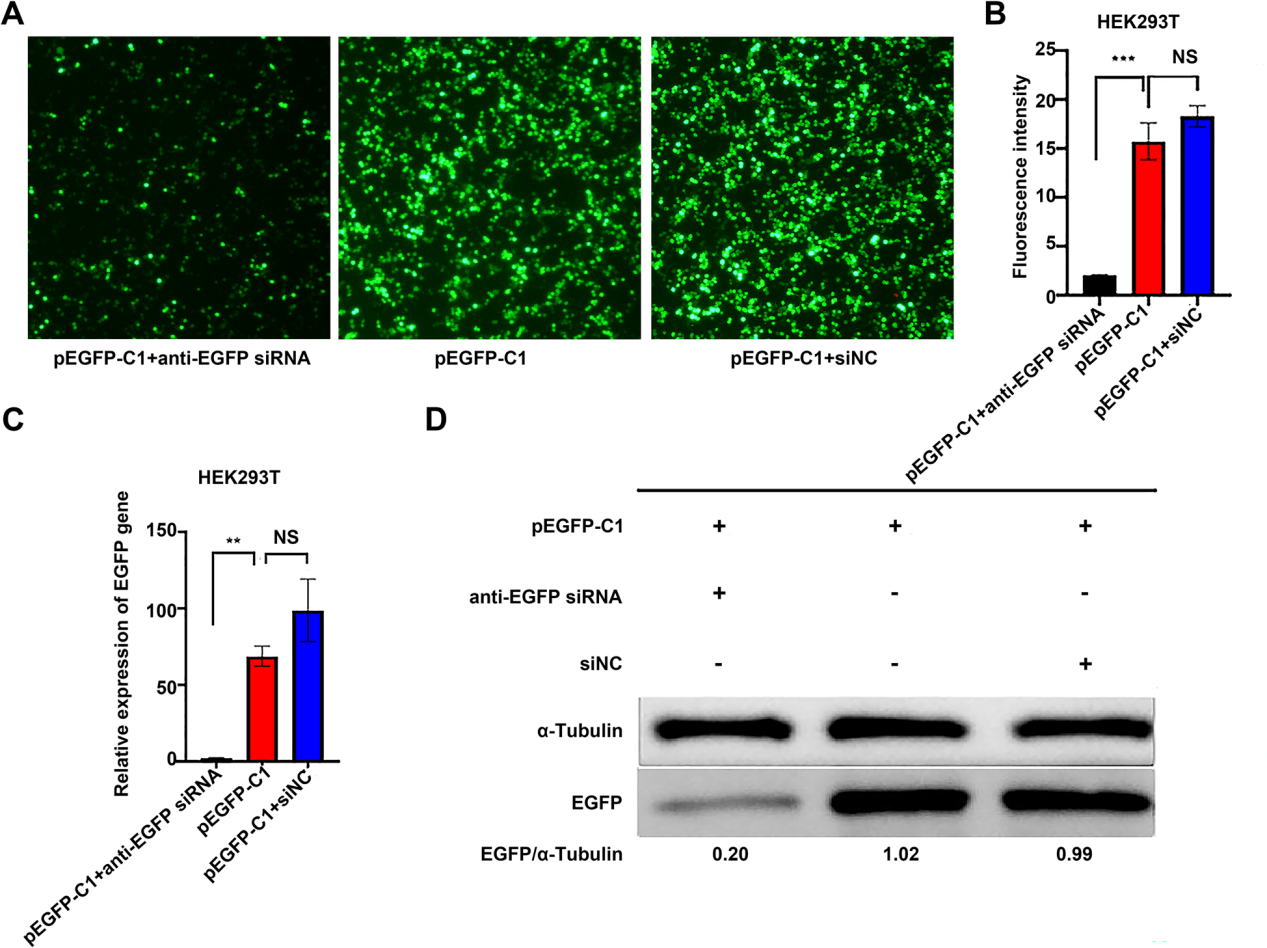
**Establishment of the siRNA-mediated EGFP-RNAi system in HEK293T cells.**

We first established the siRNA-mediated EGFP-RNAi system in HEK293T cells. HEK293T cells were transfected with a plasmid encoding EGFP together with either anti-EGFP siRNA or siNC. Then, 24 h post-transfection, EGFP fluorescence intensity was initially assessed using an inverted fluorescence microscope (Figure 2A). Subsequently, the fluorescence intensity was statistically analysed using ImageJ software (Figure 2B). Furthermore, *EGFP* mRNA levels were quantified by qRT-PCR (Figure 2C), and EGFP protein expression was analysed concurrently by western blotting (Figure 2D). The results demonstrated that compared with control groups transfected with pEGFP-C1 plasmid alone or co-transfected with pEGFP-C1 and siNC, the anti-EGFP siRNA treatment group exhibited significantly reduced levels of both EGFP mRNA and protein. These findings validate the successful establishment of a siRNA-mediated RNAi system in HEK293T cells.

### TMUV NS5 protein suppresses RNAi in HEK293T cells

**Figure 3:**
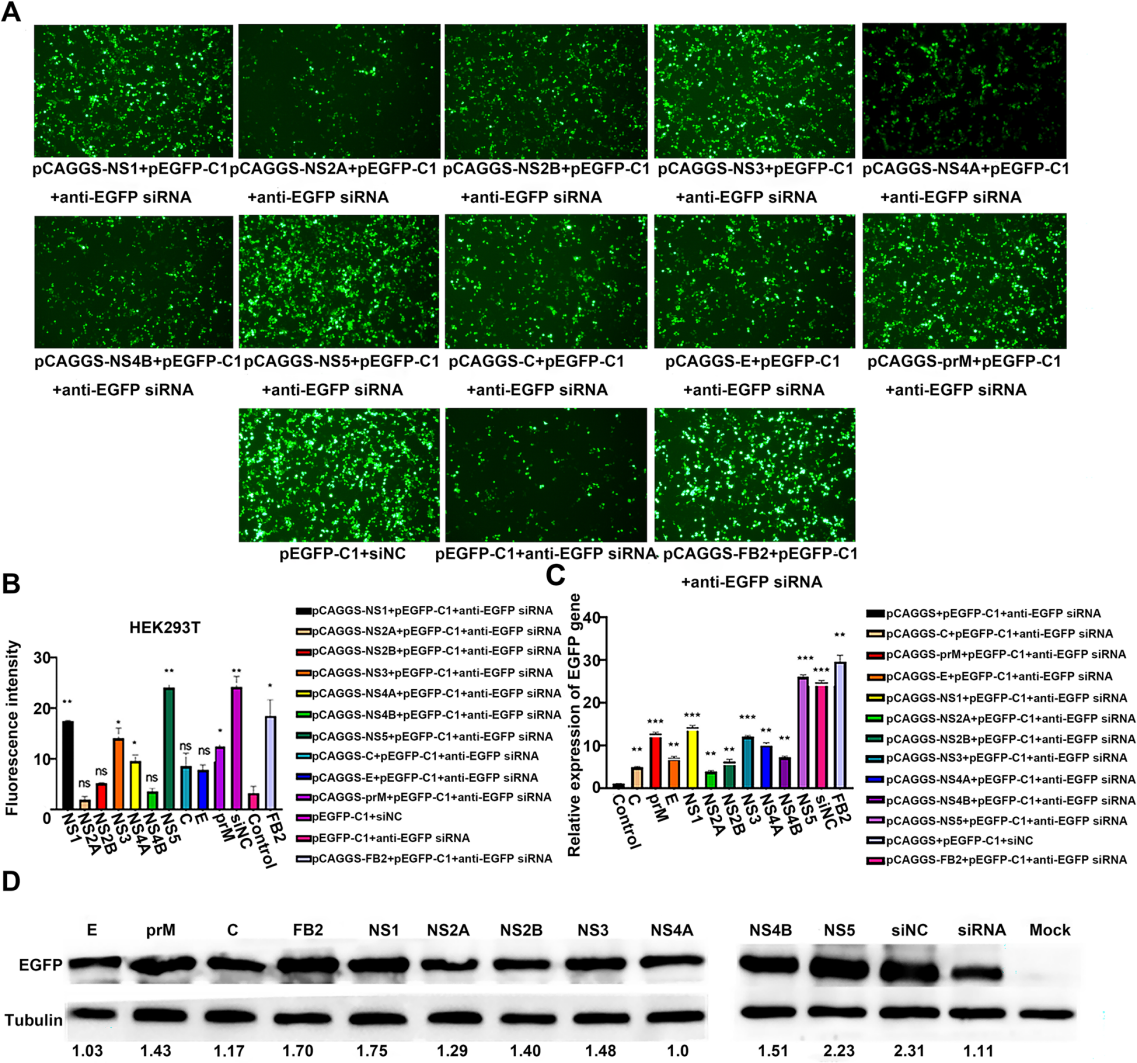
**Analysis of the impact of all TMUV proteins on siRNA-mediated RNAi.**

To investigate whether TMUV can antagonise RNAi in HEK293T cells, we first attempted to identify any TMUV proteins that could serve as potential VSRs. On the basis of the established siRNA-mediated EGFP-RNAi system, we co-transfected pEGFP-C1, anti-EGFP siRNA and TMUV protein expression plasmids into HEK293T cells. Fluorescence microscopy analysis at 48 h post-transfection revealed a significant difference in EGFP fluorescence intensity between the siNC control group and the anti-EGFP siRNA knockdown group, with the former exhibiting markedly stronger fluorescence signals (Figure 3A). Furthermore, compared with the siRNA knockdown group, significant restoration of EGFP fluorescence levels was observed in the FB2-positive control group (expressing a known viral suppressor) and in cells expressing the NS1, NS3, NS5 and prM proteins. In contrast, no significant restoration was detected in cells expressing other TMUV proteins. Statistical analysis of fluorescence intensity across all groups (Figure 3B) demonstrated that the fluorescence signal intensity was ranked as follows: siNC control group > NS5 experimental group > FB2 positive control group > NS1 > NS3 > prM > NS4A experimental group. Notably, both the NS5 experimental group and the siNC control group exhibited significantly higher fluorescence intensity than the siRNA knockdown group (*P* < 0.01). Subsequent qRT-PCR analysis (Figure 3C) confirmed these findings, showing a highly consistent trend in *EGFP* mRNA levels: mRNA levels in the NS5, NS1, NS3 and prM experimental groups were significantly elevated compared with the siRNA knockdown group (*P* < 0.001). Western blotting results (Figure 3D) further corroborated these observations, indicating the highest EGFP protein expression in the siNC group, followed by the NS5 protein expression group. Collectively, these results demonstrate that the NS5 protein of TMUV exhibits the most potent restoration of EGFP expression levels. Its RNAi antagonistic activity is significantly stronger than that of other structural and non-structural proteins tested. These findings provide preliminary evidence that NS5 is a VSR encoded by TMUV.

### Establishment of the dsRNA-mediated EGFP-RNAi system in S2 cells

**Figure 4:**
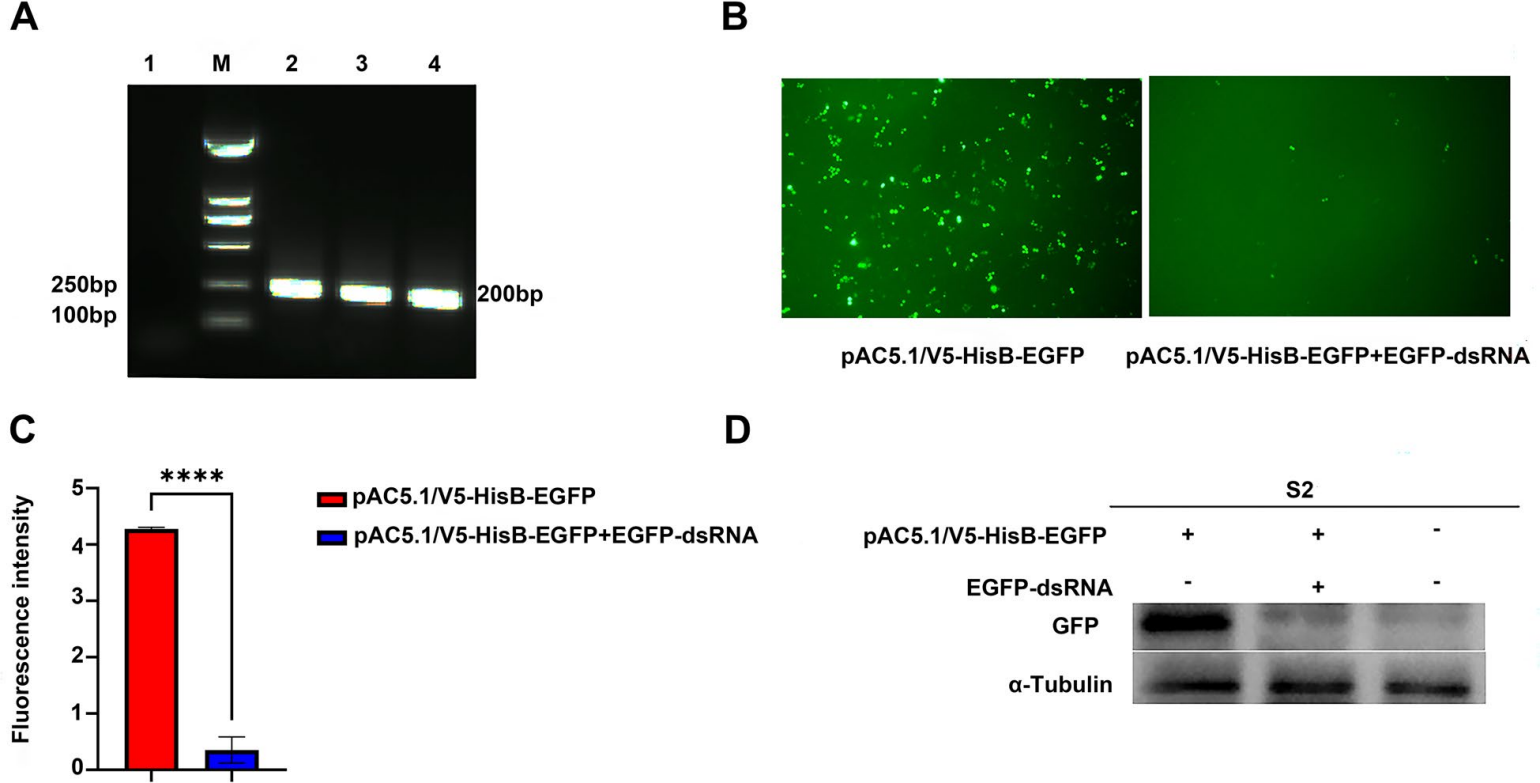
**Establishment of the dsRNA-mediated EGFP-RNAi system in S2 cells.** **A** Gel electrophoresis results after in vitro transcription of dsRNA. 1, negative control; M, DL2000 Marker; 2–4, 200 bp dsRNA. **B**–**D** EGFP-dsRNA significantly inhibits the expression of EGFP.

*Drosophila melanogaster* serves as a classical model organism for studying the RNAi pathway. Therefore, we established the dsRNA-mediated EGFP-RNAi system in S2 cells. Initially, a 200 bp EGFP-dsRNA was successfully synthesised by in vitro transcription (Figure 4A). Subsequently, the dsRNA was co-transfected with the pAC5.1/V5-HisB-EGFP plasmid into S2 cells, and using cells transfected with pAC5.1/V5-HisB-EGFP alone as the control group. Observation via inverted fluorescence microscopy revealed a marked reduction in EGFP fluorescence signals in the dsRNA-treated group compared with the control group (Figure 4B). Further quantification and statistical analysis of fluorescence intensity across groups demonstrated a highly significant difference between the control and dsRNA-treated groups (*P* < 0.001) (Figure 4C). Consistent with these findings, western blotting analysis (Figure 4D) confirmed significantly lower EGFP protein expression levels in the dsRNA-treated group relative to controls. Collectively, these results indicate that EGFP-dsRNA effectively activates the RNAi pathway in S2 cells, achieving potent silencing of the EGFP transgene and significantly suppressing EGFP protein expression.

### TMUV NS5 protein suppresses RNAi in S2 cells

**Figure 5:**
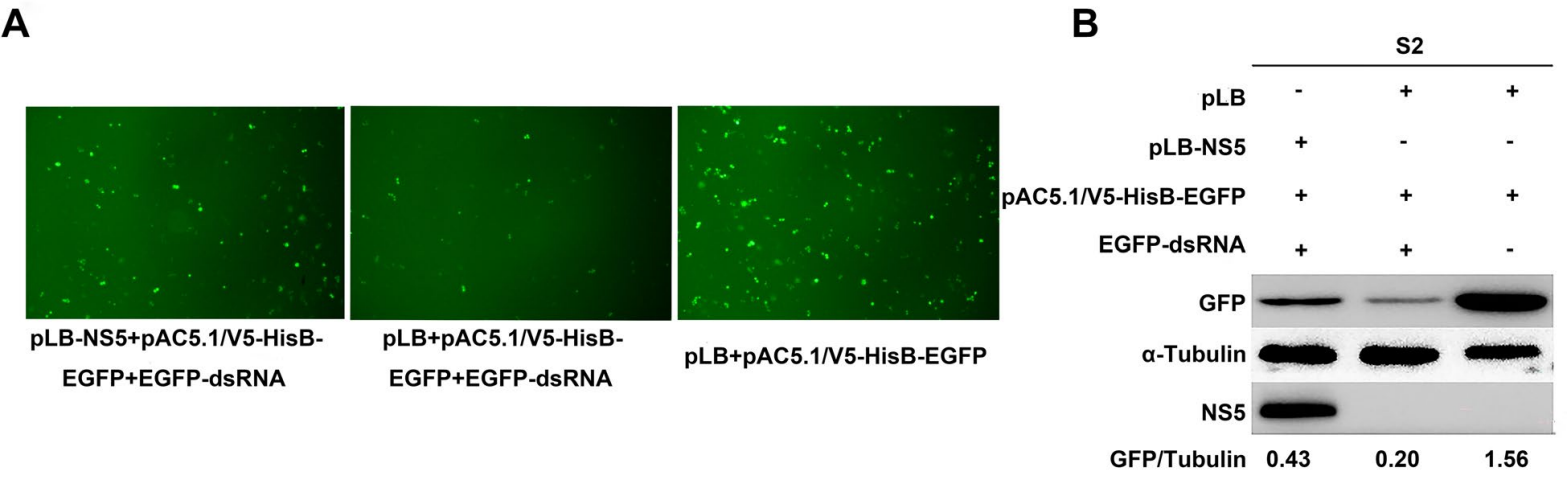
**The effect of TMUV-NS5 on dsRNA-mediated RNAi.**

To investigate whether TMUV NS5 protein can antagonise RNAi activity in S2 cells, we utilised the established dsRNA-mediated EGFP-RNAi system in *Drosophila* cells. Post-transfection analysis by inverted fluorescence microscopy revealed significantly reduced EGFP fluorescence intensity in EGFP-dsRNA-transfected cells compared with controls. In contrast, cells co-transfected with the NS5 protein exhibited substantial restoration of fluorescence levels (Figure 5A). Western blotting analysis further demonstrated that whereas dsRNA significantly suppressed EGFP protein expression, NS5 co-transfection partially restored EGFP expression (Figure 5B). These findings indicate that: (1) EGFP-dsRNA effectively activates the RNAi pathway to silence EGFP expression in S2 cells, and (2) TMUV-NS5 protein antagonises RNAi-mediated suppression, restoring EGFP protein expression. Collectively with our prior results in HEK293T cells, these data demonstrate that TMUV-NS5 possesses evolutionarily conserved VSR functionality, effectively antagonising RNAi in both *Drosophila* S2 cells and human-derived HEK293T cells.

### Interaction between NS5 protein and the RNAi pathway-associated factor DDX3X

**Figure 6:**
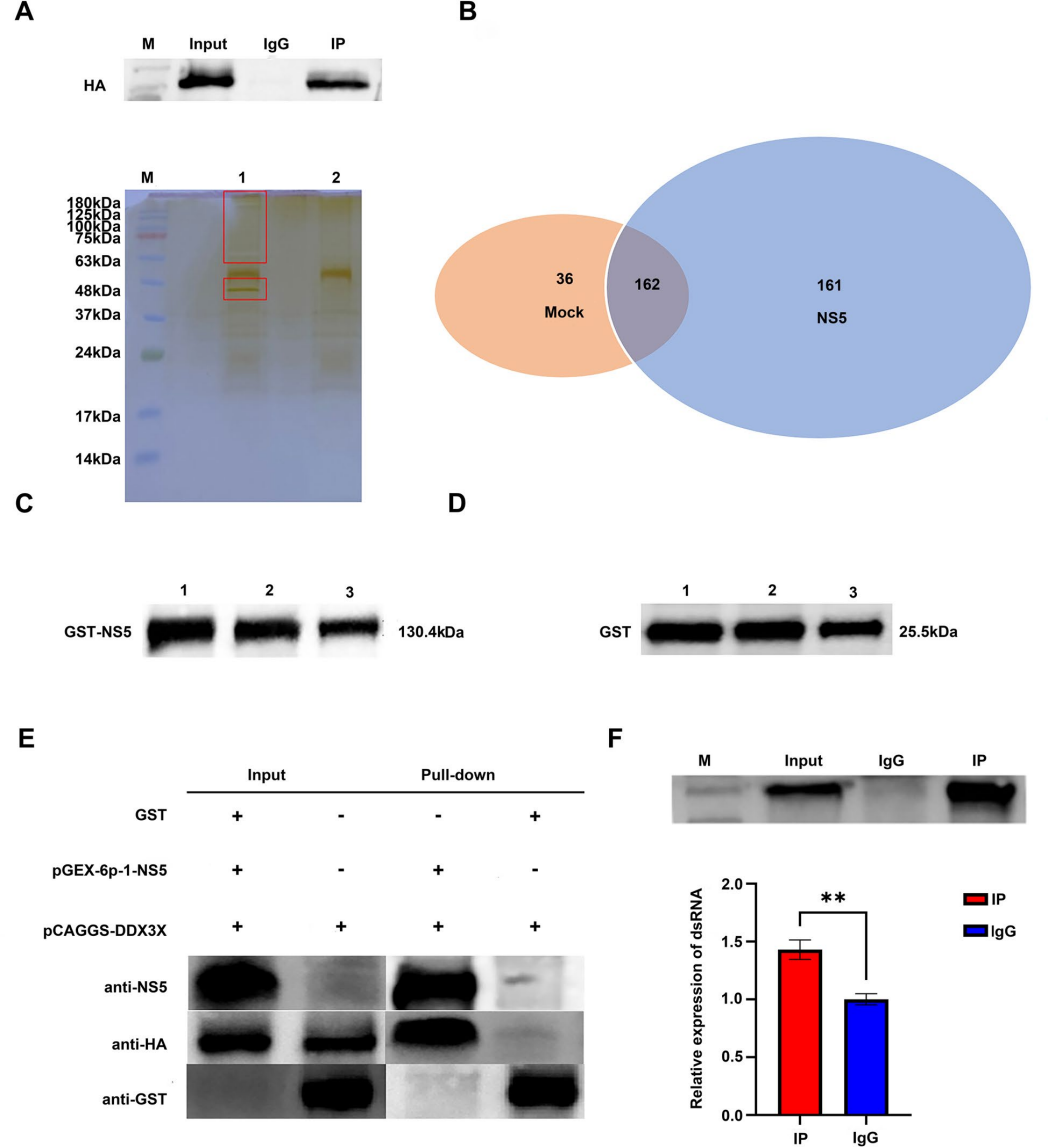
**The mechanism of TMUV NS5 protein antagonising RNAi.** **A** The results of Co-IP products. M, protein molecular weight marker; 1, experimental group; 2, control group. **B** Venn diagram of TMUV NS5 protein Co-IP mass spectrometry results. **C** Prokaryotic expression of pGEX-6p-1-NS5. 1, total bacterial protein; 2, soluble supernatant protein; 3, inclusion body protein. **D** Prokaryotic expression of pGEX-6p-1. 1, total bacterial protein; 2, soluble supernatant protein; 3, inclusion body protein. **E** GST pull-down in vitro validation of NS5 and DDX3X interaction. **F** RNA-IP assay verifies that dsRNA can interoperate with NS5 protein.

VSRs typically antagonise the host RNAi pathway through two primary mechanisms: first, they bind to key RNAi effector proteins (such as Dicer and AGO family proteins) to inhibit their activity; second, they directly bind to dsRNA to inhibit RNAi activity. To determine whether TMUV-NS5 employs the former mechanism, we first screened for host proteins potentially interacting with TMUV-NS5. The pCAGGS-NS5 plasmid was transfected into HEK293T cells, and Co-IP experiments were performed 48 h later. Protein complexes were separated by SDS–PAGE, with silver staining revealing multiple distinct bands in the NS5 antibody IP group compared with the IgG control group (Figure 6A). Differential protein bands were excised and subjected to liquid chromatography–tandem mass spectrometry (LC–MS) identification by Qingdao OE Biotech Co., Ltd. Mass spectrometry analysis identified 161 host proteins potentially interacting with TMUV-NS5 (Figure 6B). On the basis of functional relevance to viral replication, 14 key proteins were prioritised: MYH9, DDX3X, DDX17, PARP1, ADD1, ILF2, HSP90AB1, HNRNPA2B1, PCBP2, SYNCRIP, KPNB1, DHX30, TRIM28 and PABPC1 (Table 1). Notably, previous studies have confirmed that DDX3X, as a translation-essential factor of the Dicer-binding partner PACT, is not only a critical regulatory protein in the shRNA-induced RNAi process but also a direct interactor with the AGO2 protein. Given DDX3X’s pivotal role in RNAi pathways and its putative interaction with NS5, we selected it as the primary target for subsequent validation.

**Table 1:** **Potentially interacting proteins with TMUV-NS5**

Description	Gene symbol	Coverage (%)	Peptides (count)
Myosin-9	MYH9	65	137
ATP-dependent RNA helicase DDX3X	DDX3X	5	2
Probable ATP-dependent RNA helicase DDX17	DDX17	4	2
Poly(ADP-ribose) polymerase 1	PARP1	24	19
Alpha-adducin	ADD1	11	4
Interleukin enhancer-binding factor 2	ILF2	14	4
Heat shock protein HSP 90-beta	HSP90AB1	9	5
Heterogeneous nuclear ribonucleoproteins A2/B1	HNRNPA2B1	14	4
Poly(rC)-binding protein 2	PCBP2	10	3
Heterogeneous nuclear ribonucleoprotein Q	SYNCRIP	5	3
Importin subunit beta-1	KPNB1	3	2
ATP-dependent RNA helicase DHX30	DHX30	2	2
Transcription intermediary factor 1-beta	TRIM28	2	2
Polyadenylate-binding protein 1	PABPC1	5	3

Subsequently, we further verified the interaction between the NS5 protein and DDX3X through a GST pull-down experiment. We first induced the expression of the GST-NS5 fusion protein and GST tag protein at 16 °C for 18 h. Findings revealed the successful expression of both the GST-NS5 fusion protein and the GST tag protein, with greater levels of expression in soluble segments compared with inclusion bodies (Figures 6C and D). Soluble GST-tag and GST-NS5 proteins from prokaryotic expression were immobilised on GST-tagged resin, serving as the control and experimental groups, respectively. Then the eukaryotic-expressed prey protein DDX3X was added to both groups and incubated together. Following incubation, bait–prey complexes were eluted, and protein loading buffer was added to denature the proteins.

Western blotting analysis (Figure 6E) showed that the experimental group detected the GST-tagged NS5 bait protein with a size of 130.4 kDa and the HA-tagged DDX3X prey protein with a size of 73 kDa. In contrast, the control group only detected a GST tag protein with a size of 25.5 kDa and did not detect the DDX3X protein. The results indicate that the bait protein GST-NS5 and the prey protein HA-DDX3X can interact in vitro.

### The dsRNA-binding activity of TMUV-NS5 protein

To further determine whether TMUV-NS5 exerts its VSR function through direct dsRNA binding, HEK293T cells were co-transfected with 15 μg pCAGGS-NS5 plasmid and 7.5 μg of 200 bp EGFP-dsRNA as the experimental group. Control cells were co-transfected with equivalent amounts of pCAGGS empty vector plasmid and dsRNA. After 48 h, cell samples were collected, and RNA-IP and qRT-PCR analysis revealed (Figure 6F) that the level of captured dsRNA in the experimental group was significantly higher than that in the control group (*P* < 0.01). These results indicate that TMUV-NS5 protein specifically binds dsRNA in mammalian cells to antagonise host RNAi activity.

## Discussion

TMUV is one of the important pathogens that seriously endangers the waterfowl breeding industry. In-depth research on its interaction with the host is crucial for understanding the pathological process of viral diseases and developing antiviral diagnosis and treatment strategies. RNAi is a highly conserved and sequence-specific mechanism for antiviral gene silencing in all eukaryotic cells. However, numerous viruses have evolved evasion strategies, encoding VSRs to counteract the host’s RNAi immune response. This study demonstrates that TMUV infection of mammalian cells initially induces the robust production of vsiRNAs and an effective antiviral RNAi response during the early stages of infection. Notably, as infection time increases, TMUV effectively antagonises the effect of RNAi. Specifically, the structural protein NS5, encoded by TMUV, has been identified as possessing VSR activity, which can competitively bind to dsRNA with the Dicer protein, thereby inhibiting the generation of siRNA mediated by Dicer. Furthermore, the NS5 protein interacts with DDX3X, a protein essential for the translation of PACT, a binding partner of Dicer.

Previous studies have demonstrated that infections by diverse viruses, such as Sindbis virus (SINV), Nodamura virus (NoV), Zika virus (ZIKV) and encephalomyocarditis virus (EMCV), can induce robust production of vsiRNAs in host cells [21, 22]. The production of these vsiRNAs is a key marker of host RNAi antiviral immune activation. This study demonstrates that TMUV infection of HEK293T cells similarly induces substantial vsiRNAs generation at 12 hpi. This finding indicates that TMUV effectively activates the host RNAi antiviral pathway during the initial phase of infection. However, no characteristic vsiRNAs were detectable at 24 hpi. This phenomenon is consistent with relevant studies demonstrating that flaviviruses can induce and antagonise RNAi [19]. Collectively, these observations suggest that as TMUV infection progresses, the virus likely employs a comparable strategy to antagonise the host’s RNAi immune defence.

Numerous viruses have been documented to encode VSR to antagonise RNAi. Notable examples include: the nonstructural protein 7a of severe acute respiratory syndrome coronavirus (SARS-CoV) [23], the Tas protein of primate foamy virus [24] and the nonstructural protein 3A of enterovirus A71 (EV-A71) [25, 26]. In addition, examples of *Flavivirus* include: the sfRNA of Zika virus (ZIKV) [27], the capsid protein of yellow fever virus (YFV) [28] and the nonstructural protein NS2A, NS4B and NS3 of the dengue virus (DENV) [18, 19, 29]. Although all flaviviruses exhibit inherent RNAi-antagonising activity, VSRs are not evolutionarily conserved across different members of the genus *Flavivirus*. Furthermore, some flaviviruses, such as DENV, even have multiple VSRs to synergistically counteract host RNAi-mediated antiviral defence. This study demonstrates that the TMUV NS5 protein also has VSR activity. NS5 significantly suppresses the RNAi-mediated antiviral response in both HEK293T cells and *Drosophila* S2 cells. This finding thus establishes the NS5 protein as a potential target for the development of broad-spectrum antiviral therapeutics.

The VSR protein encoded by viruses can evade RNAi through multiple mechanisms. For instance, the E2 protein of the hepatitis C virus (HCV) antagonises RNAi by interacting with the host AGO2 protein [20]. The B2 protein of Nodamura virus (NoV) and the 3A protein of enterovirus A71 (EV-A71) prevent Dicer from cutting dsRNA to produce siRNA by directly chelating dsRNA [30]. Similarly, the P126 protein of tomato bushy stunt virus (TBSV) inhibits its 3′-end methylation by binding to siRNA, resulting in siRNA degradation [31]. This study reveals that the TMUV NS5 protein antagonises RNAi through a dual mechanism: (1) direct binding to dsRNA, and (2) interaction with DDX3X, a critical protein component of the RNAi pathway. Given the core role of DDX3X in PACT-mediated Dicer function and AGO2 recruitment [32], NS5 likely hijacks this pivotal regulatory node to antagonise the host RNAi response.

Nevertheless, critical questions remain to be elucidated. First, the specific domain responsible for the VSR activity of the TMUV NS5 protein has not yet been identified. This necessitates screening through the construction of truncated mutants, followed by site-directed mutagenesis to pinpoint critical amino acid residues. In addition, the precise molecular mechanism by which NS5 antagonises RNAi through interaction with DDX3X requires further investigation.

To our knowledge, this study is the first to confirm that TMUV infection of mammalian cells induces robust production of vsiRNAs and elicits a potent antiviral RNAi response during the early stages. Subsequently, however, the virus antagonises this response by encoding its NS5 protein, which exhibits VSR activity. Furthermore, we have delineated the dual molecular mechanisms by which the TMUV NS5 protein antagonises the RNAi pathway. These findings establish a crucial theoretical foundation for elucidating immune evasion strategies for TMUV and developing RNAi-based TMUV prevention and control technologies.

## Data Availability

All data generated or analysed during this study are included in this article.
